# Hypoxic response elements and Tet-On advanced double-controlled systems regulate *hVEGF*_165_ and *angiopoietin-1* gene expression *in vitro*^☆^

**DOI:** 10.1016/S1674-8301(11)60027-4

**Published:** 2011-05

**Authors:** Hao Zhang, Hongyan Dong, Bo Jiang, Zheng Wang, Rui Chen, Zhifeng Zhang, Zhongming Zhang

**Affiliations:** a Institute of Cardiovascular Disease, Affiliated Hospital of Xuzhou Medical College, Xuzhou, Jiangsu 221002, China;; b Center of Biological Research, Xuzhou Medical College, Xuzhou, Jiangsu 221002, China.

**Keywords:** gene control, vascular endothelial growth factor, angiopoietin-1, hypoxia, doxycycline, cardiomyocyte

## Abstract

Angiogenesis in ischemic tissue is a complex and multi-gene event. In the study, we constructed hypoxic response elements (HRE) and the Tet-On advanced double-controlled systems and investigated their effects on the expression of *hVEGF_165_* and *angiopoietin-1* (*Ang-1*) genes in rat cardiomyocytes exposed to hypoxia and pharmacologic induction. We infected neonatal rat cardiomyocytes with recombinant rAAV-rtTA-Rs-M2/rAAV-TRE-Tight-Ang-1 and rAAV-9HRE- hVEGF_165_. Our results indicated that the viral titer was 1×10^12^ vg /mL and the viral purity exceeded 98%. *hVEGF*_165_ expression was induced by hypoxia, but not by normoxia (*P* < 0.001). *Ang-1* expression was evident under doxycycline induction, but undetectable without doxycycline induction (*P* < 0.001). Immunofluorescence staining showed that positively stained hVEGF_165_ and Ang-1 protein appeared only under both hypoxia and doxycycline induction. We demonstrate here that HRE and the recombinant Tet-On advanced double gene-controlled systems sensitively regulate the expression of *hVEGF_165_* and *Ang-1* genes in an altered oxygen environment and under pharmacological induction *in vitro*.

## INTRODUCTION

Therapeutic angiogenesis is a therapeutic intervention that introduces exogenous angiogenic growth factors to enhance collateral blood flow in the infarcted myocardium and improve global heart performance[1],[2]. Although increasing interest in therapeutic angiogenesis has focused on vascular endothelial growth factor (VEGF), one of the most potent angiogenic factors, some studies indicate that VEGF, as an early-phase angiogenic factor, often produces immature vessels that are unable to create functional collateral development[3]–[5]. Moreover, uncontrolled long-term expression of VEGF delivered by recombinant adeno-associated virus (rAAV) vector *in vivo* may result in side effects such as hemangioma formation, retinopathy or arthritis[6].

It is well documented that angiogenesis is a complex event in which multiple angiogenic factors may exert their actions at different phases of angiogenesis[7]. Therefore, for therapeutic angiogenesis, in addition to early angiogenic factors, introduction of late phase angiogenic factors to modify immature vasculature and enhance neovessel function has attracted considerable interest. Data indicate that angiopoietin-1 (Ang-1) plays a critical role in promoting maturation of VEGF-induced vessels in the late phase of angiogenesis[8]. However, uncontrolled early Ang-1 expression in ischemic tissues is likely inadequate to remodel VEGF-induced capillaries and does not benefit functional angiogenesis[9],[10]. Clinically, due to limitations in gene delivery technology, multiple genes are usually transferred simultaneously. However, expression of multiple angiogenic genes at the same time is not consistent with the physiology of angiogenesis. Therefore, timely, controlled expression of these genes *in vivo* is required. Thus far, the ideal multi-gene expression control system in ischemic heart is still under development.

Our preliminary experiment confirmed that the hypoxic response element (HRE), as an effective gene switch, reliably induced the expression of the *hVEGF_165_* gene expression under hypoxia and re-oxygenation both *in vivo* and *in vitro*[11]–[13]. In the present study at the cellular level, we employed recombinant Tet (tetracycline)-On together with the HRE system and investigated the feasibility of regulated expression of *hVEGF*_165_ and *Ang*-*1* genes in cardiomyocytes in an altered oxygen environment and under pharmacological induction. We aimed to explore a new approach of therapeutic gene control for further selected multiple gene therapy in ischemic heart disease *in vivo*.

## MATERIALS AND METHODS

### Tissues and animals

A healthy adult lung was obtained from the National Human Genome Center in Shanghai, China. Total human RNA was extracted using TRIzol Reagent (Invitrogen, USA).

Neonatal Sprague-Dawley rats (1-3 d old, weighing 5-7 g, mean 6.3±0.6 g) were obtained from The Experimental Animal Centre of Xuzhou Medical College. The study protocol was approved by the local Institutional Review Board at the authors affiliated institution. Acquisition of human tissue specimens was carried out in accordance with the institution guide line. All animals received humane care in compliance with the Guideline for Care and Use of Laboratory Animals published by Jiangsu Province, China.

### Expression vectors and plasmid construction

The rAAV-9HRE-*h*VEGF_165_ plasmid was a gift from Dr. Hua Su (the Cardiovascular Research Institute, University of California, USA). Recombinant AAV vectors were prepared with a three-plasmid cotransfection system as described previously[13],[14]. The titer of rAAV-9HRE-*h*VEGF_165_ was 2×10^12^ vector genomes (vg/mL).

For the Tet-On advanced system, a reverse Tet Transactivator (rtTA)-Rs-M2, a p-tetracycline response element (pTRE)-Tight, a pTRE-Tight-luc and AAV expression vectors (pSNAV) were purchased from Clonetech, Inc. (USA) and Vector Gene Technology Ltd (Beijing, China). All vector constructs were confirmed by DNA sequencing.

Briefly, total RNA from human lung tissue was isolated using TRIzol Reagent (Invitrogen, USA); 2 µg of total RNA was reverse-transcribed with SuperScript™ III Reverse Transcriptase (Invitrogen, USA) according to the manufacturer's instructions. PCR amplification was performed using the cDNA isolated from total cellular RNA with the PrimeSTAR™ HS DNA polymerase kit (TaKaRa, Japan). The primers for first-round PCR were F0: 5′-ACGGTCACACAGAGAGGAAACA-3′ and R0: 5′-CAGCTTCTCCGGATTTCTTTGT-3′. The primers for second-round PCR were Ang-1-F: 5′-GCGGATCCATGACAGTTTTCCTTTCC-3′ and Ang-1-R: 5′-GCAAGCTTTCAAAAATCTAAA GGTCG-3′, which were designed based on the sequence of the *Ang*-*1* gene (GenBank, No. NM 001146) and were synthesized by Invitrogen, USA.

To facilitate cloning, F and R primers contained a *Bam*HI site at the 5′ end of the coding sequence of F and a *Hin*dIII site at the 5′ end of R (underlined above). After being recovered from the gel using an agarose gel DNA purification kit (TaKaRa, Japan), the PCR-amplified DNA was added with a poly “A” tail with the DNA A-tailing kit (TaKaRa, Japan) and ligated into the pMD18-T simple vector (TaKaRa, Japan). The primary structure of the insert was confirmed by direct sequencing. The fragment of coding *Ang*-1 was released from the pMD18-T-Ang-1 by digestion with *Bam*HI and *Hin*dIII and subcloned into the expression vector pTRE-Tight as described previously[15]. The obtained recombinant eukaryotic expression vector pTRE-Tight-Ang-1 was identified by PCR and then analyzed by the restriction enzymes *Bam*HI/*Hin*dIII.

The fragments encoding rtTA-Rs-M2 (765bp), TRE-Tight-Ang-1 (1.8kb) and TRE-Tight-Luc (2kb) were released, respectively, from pTet-On advanced, pTRE-Tight-Ang-1 and pTRE-Tight-Luc by digestion with *Eco*RI/*Bgl* II, *Xho*I/*Sal*I or *Xho*I/*Nhe*I. Afterwards, they were ligated to *Eco*RI/*Bgl* II, *Xho*I/*Sal*I or *Xho*I/*Nhe*I sites of the empty vectors (pSNAV), respectively. The recombinant eukaryotic expression vectors pSNAV-rtTA-Rs-M2, pSNAV- Tight-Ang-1 and pSNAV-TRE-Tight-Luc were identified by PCR and digested with *Eco*RI/*Bgl* II, *Xho*I/*Sal*I or *Xho*I/*Nhe*I, respectively.

### Viral production determination

Three plasmid constructs (rtTA-Rs-M2, pTRE-Tight, pTRE-Tight-Luc) were transfected into BHK-21 cells in six-well plates with Lipofectamine 2000 (Invitrogen, USA). The cells were maintained with G418 (800 µg/mL) for 24 h. Three cell lines were designated as BHK/Tet-On, BHK/TRE-Tight-Ang-1 or BHK/TRE-Tight-Luc and were infected with HSV1-rc/△UL2 at a multiple of infection (MOI) of 0.1 for 48 h until all the cells exhibited cytopathic effect. The PEG/NaCl precipitated chloroform extraction technique was used to purify the vectors of rAAV-rtTA-Rs-M2, rAAV-TRE-Tight-Ang-1 and rAAV-TRE-Tight-Luc.

The purified plasmids were used as standards, and the probes were labeled by PCR. The serially diluted virus stock suspension and standards preparations were determined by the dot-blot method using digoxigenin-labeled gene as probe. A viral titer was generated from the detected hybridization signals. The protein traps were obtained from 10% SDS-PAGE electrophoresis and the viral purity was also determined.

### Cardiomyocyte culture and identification

Following ether inhalation anesthesia, thoracotomy was performed. The animal heart was rapidly collected, cut into small pieces and digested with trypsin (1.25 mmol/L) for 10 min. The separated cells were centrifuged at 200 *g* for 7 min and then suspended in DMEM for 2 h (1% CO_2_ and 99% air). To inhibit fibroblast growth, unattached cells were treated with 5-bromodeoxyuridine (Brdu, 25 mmol/L) for 24 h before infection and then maintained in DMEM supplemented with 10% FBS.

For determination of cellular purity, cardiac troponin-I (cTnI) staining was completed with a rabbit polyclonal antibody against cTnI (Chemicon, USA) and secondary goat anti-rabbit IgG antibody (Sigma, USA). After hematoxylin and eosin (HE) staining, cardiomyocytes chosen randomly in six visual fields were visualized under a light microscope. The number of cTnI positively stained cells (N2) and nuclei with HE positive staining (N1) were counted. Cardiomyocyte purity was determined based on the following formula: cellular purity = N2/N1×100%.

### Viral infection and treatments

To assay the optimal proportion of the virus for Ang-1 transfection, rAAV-rtTA-Rs-M2 was mixed with a fixed amount of rAAV-TRE-Tight-Ang-1 (10^10^ vg) in different ratios (1:1, 1:2, 1:3, 1:4, 1:5 and 1:6). After 12 h infection, the cells were treated with 1 µg/mL doxycycline hydrochloride (Dox, Sigma, USA) for 12 h.

An optimal Dox concentration for Ang-1 expression induction was determined. rAAV-rtTA-Rs-M2 and rAAV-TRE-Tight-Ang-1 at a ratio of 1:4 were infected into the cultured cells (10^10^ vg). Ang-1 expression was induced by addition of Dox at different concentrations (0.01-10.0 µg/mL). The cells were harvested after 12 h incubation.

For the determination of an optimal concentration of rAAV-TRE-Tight-Ang-1 for infection, the cells were infected with different concentrations of rAAV-TRE-Tight-Ang-1 (10^11^, 5×10^10^, 10^10^, 5×10^9^ and 10^9^) for 12 h. Then, the cells were treated with 1 µg/mL Dox for 12 h, 10^10^ vg was also used as a control without Dox induction. Similarly, to identify the optimal concentration of rAAV-9HRE-*h*VEGF_165_, the cells were infected with different concentrations of rAAV-9HRE-*h*VEGF_165_ (10^11^, 5×10^10^, 10^10^, 5×10^9^, and 10^9^ vg and PBS) for 8 h and then cultured in a hypoxic incubator (1% O_2_) for an additional 12 h[12].

The cardiomyocytes were divided into eight groups. Group A: The cells were infected by rAAV-9HRE-hVEGF_165_ and cultured under hypoxia (1% O_2_). Group B: The cells were co-infected with rAAV-9HRE-hVEGF_165_ and rAAV-rtTA-Rs-M2 / rAAV-TRE-Tight-Ang-1 (1:4) under hypoxia without Dox induction. Group C: The cells were co-infected with rAAV-9HRE-hVEGF_165_ and rAAV-rtTA-Rs-M2 / rAAV-TRE-Tight-Ang-1 under hypoxia with Dox induction (1 µg/mL). Group D: The cells were co-infected with rAAV-9HRE-*h*VEGF_165_ and rAAV-rtTA-Rs-M2 / rAAV-TRE-Tight-Ang-1 under normoxia with Dox induction. Group E: The cells were infected with rAAV-rtTA-Rs-M2 / rAAV-TRE-Tight-Ang-1 under normoxia with Dox induction. Group F: The cells were infected with rAAV-9HRE-Laz under hypoxia, as vector control. Group G: The cells were infected with rAAV-rtTA-Rs-M2 and rAAV-TRE-Tight-Luc under normoxia with Dox induction, as vector control. Group H: An equal volume of PBS was added to the cells under hypoxia with Dox induction, as blank control.

### Western blotting studies

The cells were solubilized in lysis buffer (100 mmol/L Tris-HCl, 4% SDS, 20% glycerine, 200 mmol/L DTT and protease inhibitors, pH 6.8). Total cellular protein was denatured by boiling for 10 min with an equal volume of 2×Tris-glycine SDS buffer. Protein was separated by 10% SDS-PAGE and transferred to nitrocellulose membrane (Millipore, USA). After blocking with 5% non-fat milk/PBS-T for 3 h at room temperature, the membranes were incubated with a goat anti-Ang-1 antibody (Santa Cruz, USA) and a mouse anti-hVEGF_165_ antibody (Sigma, USA), respectively. Then, fluorescently labeled secondary antibody (Rockland, USA) was added for 1 h and subsequently scanned by the Odyssey Infrared Imaging System (Li-Cor Biosciences, USA).

### RT-PCR

Total RNA from cultured cells was extracted using RNAiso Reagent. RT-PCR was performed by using 1 µg of total RNA as described by the M-MLV (RNase H) procedure. The primer sequences of hVEGF165 were 5′-CTTGCCTTGCTGCTCTACCT-3′ for the forward primer and 5′-CCTTGCAACGCGAGTCTGT -3′ for the reverse primer. The primer sequences of Ang-1 were 5′-GCGGATCCATGACAGTTTCCTTTCC-3′ for the forward primer and 5′-GCAAGCTTTCAAAAATCTAAAGGTCG-3′ for the reverse primer.

### Immunofluorescence analysis

Total mixed virus (rAAV-9HRE-hVEGF_165_ and rAAV-rtTA-Rs-M2 / rAAV-TRE-Tight-Ang-1, at 1:1 ratio, 4×10^10^ vg) was added to FBS-free culture medium for 6 h. Then, the cells were cultured under hypoxic conditions (1% O_2_) for 12 h. The cells were then treated with 1 µg/mL Dox under re-oxygenation (95% air / 5% CO_2_) up to 12 h. The harvested cells were stained with a mouse anti-hVEGF_165_ antibody (Sigma, USA) and a goat anti-Ang-1 antibody (Santa Cruz, USA) at 4°C for 48 h. The secondary antibodies, conjugated with fluorescein isothiocyanate for VEGF (a goat anti-mouse FITC fluorescent antibody 1:150, Sigma, USA) or rhodamine for Ang-1 (a rabbit anti-goat FITC fluorescent antibody, Boster LTD, China), were added. The cells were observed under a fluorescence microscope (Olympus, Japan).

### Statistical analysis

All data are expressed as mean±SD and analyzed by one-way ANOVA and Student-Newman-Keuls test. *P* values less than 0.05 were considered statistically significant.

## RESULTS

### Recombinant virus identification and cardiomyocyte purity determination

**Fig. 1:**
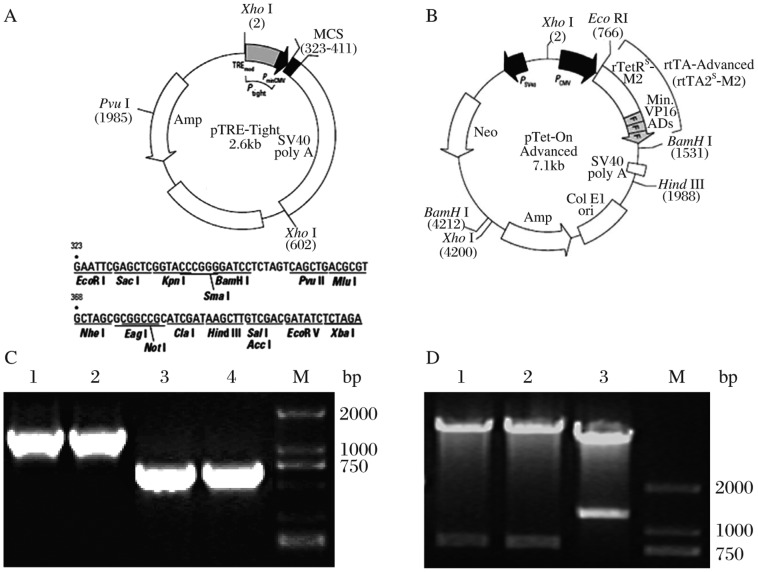
Identification of the recombinant Tet-On advanced system. Structures of the Tet-On advanced system for *Ang*-*1* gene expression control (A) pTRE-Tight and (B) pTet-On advanced (rtTA-Rs-M2). C: RT-PCR determination. Lanes 1 and 2: PCR product of pSNAV- TRE-Tight-Ang-1 (1.8 kb). Lanes 3 and 4: PCR product of pSNAV- rtTA-Rs-M2 (765 bp). M: DL2000 DNA marker. D: The restriction enzyme analyses. Lanes 1 and 2: pSNAV-rtTA-Rs-M2 was digested with *Eco*RI/*Bgl*II, two bands (7.1kb/765bp) were obtained. Lane 3: pSNAV- Tight-Ang-1 was digested with *Xho* I / *Sal* I, two bands (6.3 kb/1.8 kb) were obtained.

A structure of the Tet-On advanced system for Ang-1 gene expression control is shown in ***Fig. 1A***. PCR products of pSNAV-TRE-Tight-Ang-1 (1.8 kb) and pSNAV-rtTA-Rs-M2 (765 bp) are shown in ***Fig. 1B***. pSNAV-rtTA-Rs-M2 was digested with *Eco*RI/*Bgl*II and two bands (7.1 kb/765 bp) were obtained. Moreover, pSNAV-Tight-Ang-1 was digested with *Xho*I/*Sal*I and two bands (6.3kb/1.8kb) were available (***Fig. 1B***). The results of the PCR and restriction enzyme analyses indicated that pSNAV-rtTA-Rs-M2, pSNAV- TRE- Tight-Ang-1 and pSNAV-TRE-Tight-Luc were successfully constructed (***Fig. 1B and Fig. 1C***. The titer of recombinant virus was 1×10^12^ vg /ml and the viral purity of rAAV-rtTA-Rs-M2, rAAV-TRE-Tight-Ang-1 and rAAV-TRE-Tight-Luc was greater than 98%.

**Fig. 2:**
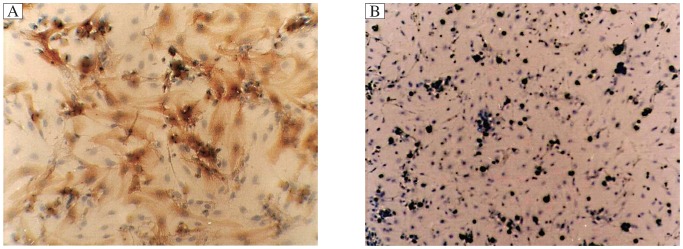
The purity of myocardial cells indicating the identification of myocardial cells. A: cTnI positive staining. B: Negative control (×200).

The representative photo for the purity of myocardial cells was provided to indicate the identification of myocardial cells (***Fig. 2***), and the purity of the cardiomyocytes was (90±3)%.

### Optimal Dox concentration for induction of Ang-1 expression

**Fig. 3:**
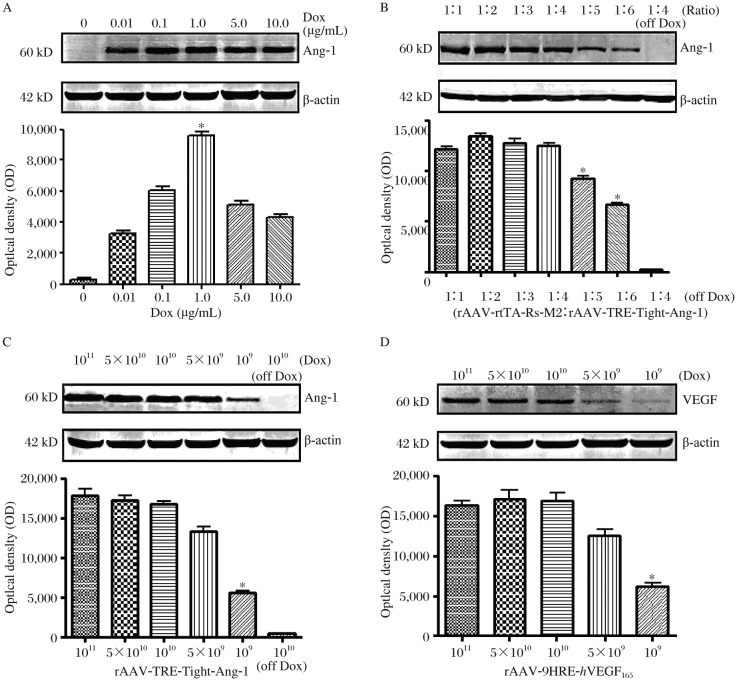
Determination of inducible gene expression of *Ang*-*1*. A: High levels of *Ang*-*1* expression were found with 1 µg/mL Dox induction. Compared with groups 0, 0.01, 0.1, 5.0 and 10.0,*P < 0.05. B: There was no significant difference among ratios of 1:1 to 1:4 (rAAV-rtTA-Rs-M2 / rAAV-TRE-Tight-Ang-1). Compared with groups 1:1, 1:2, 1:3 and 1:4, *P < 0.05. C: There was no significant difference among concentrations of 10^11^, 5×10^10^ and 10^10^ (rAAV-TRE-Tight-Ang-1). Compared with groups 10^11^, 5×10^10^ and 10^10^, *P < 0.05. D: There was no significant difference among concentrations of 10^11^, 5×10^10^ and 10^10^ (rAAV-9HRE-*h*VEGF_165_). Compared with groups 10^11^, 5×10^10^ and 10^10^, *P < 0.05.

Western blotting studies showed that a low basal level of Ang-1 protein expression was detected in the absence of Dox. A higher level of Ang-1 protein expression was achieved with 1 µg/mL Dox induction. Ang-1 expression was found in a dose-dependent manner with Dox concentration (***Fig. 3A***).

### Optimal proportion of rAAV-rtTA-Rs-M2 and rAAV-TRE-Tight-Ang-1

We found that when the ratio of rAAV-rtTA-Rs-M2 and rAAV-TRE-Tight-Ang-1 was set to 1:4, a high level of Ang-1 protein expression was achieved. Even at the optimal ratio (1:4) of rAAV-rtTA-Rs-M2 and rAAV-TRE-Tight-Ang-1, there was no Ang-1 protein expression without Dox induction (***Fig. 3B***).

### Optimal concentrations of rAAV-TRE-Tight-Ang-1 and rAAV-9HRE-*h*VEGF_165_

Under Dox induction, a high level of Ang-1 protein expression was evident when the total amount of rAAV-TRE-Tight-Ang-1 reached 10^10^ vg. Ang-1 protein expression was decreased following reduction of rAAV-TRE-Tight-Ang-1. There was no Ang-1 protein expression in the absence of Dox regardless of the amount of rAAV-TRE-Tight-Ang-1 (***Fig. 3C***). A higher level of *h*VEGF_165_ protein expression was obtained with 10^10^ vg virus (rAAV-9HRE-*h*VEGF_165_). Following decrease of rAAV-9HRE-*h*VEGF_165_, *h*VEGF_165_ protein expression was reduced simultaneously (***Fig. 3D***).

### hVEGF_165_ and Ang-1 expression

**Fig. 4:**
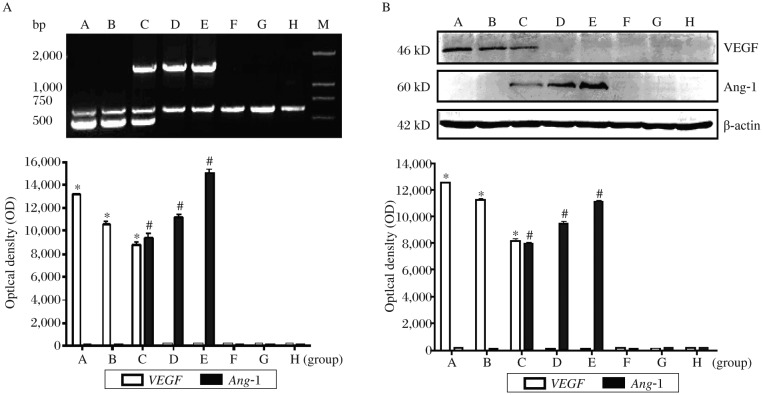
Gene expression of *hVEGF*_165_ and *Ang-1* in cardiomyocytes. A: *hVEGF*_165_mRNA (484bp) was evident in group A, B and C under hypoxia. *Ang*-*1* mRNA (1,497bp) was found in group C, D and E with Dox induction, but was undetected in the other groups. Compared with group D, E, F, G and H,*P < 0.001. Compared with group A, B, F, G and H, #*P* < 0.001. B. Expression of *h*VEGF_165_ protein was detected in group A, B and C under hypoxia. Ang-1 protein expression appeared in group C, D and E with Dox induction, but was undetected in the other groups. Compared with group D, E, F, G and H, *P < 0.001. Compared with groups A, B, F, G and H, #*P* < 0.001.

RT-PCR determination indicated that *hVEGF_165_* mRNA bands of about 484 bp were found in group A, B and C, but not in the other groups (*P* < 0.001). *Ang*-*1* mRNA bands about 1497 bp were found in group C, D and E where Dox was administered, but not identified in group F, G and H (*P* < 0.001). Both *hVEGF_165_* and *Ang*-*1* mRNA expression was found in group C only (***Fig. 4A***).

Western blotting determination indicated that *h*VEGF_165_ protein expression was detected in group A, B and C, but not in the other groups (*P* < 0.001), while Ang-1 protein expression was found in group C, D and E, but not identified in the other groups (*P* < 0.001). Both hVEGF_165_ and Ang-1 protein expression appeared in group C only (***Fig. 4B***).

**Fig. 5:**
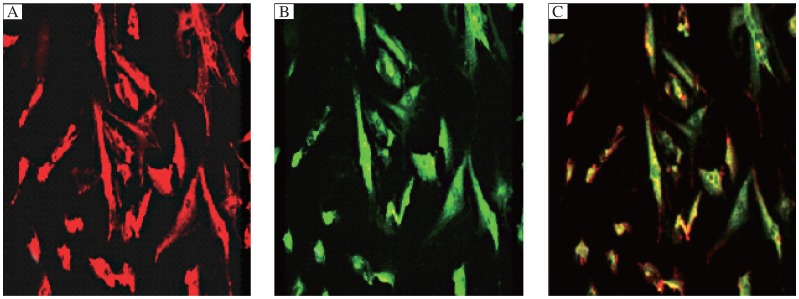
Representative figures of immunofluorescence staining. rAAV-9HRE-*h*VEGF_165_ and rAAV-(rtTA-Rs-M2/TRE-Tight-Ang-1) were co-infected into cardiomyocytes in group C under hypoxia and Dox induction. A: Red fluorescence (Ang-1). B: Green fluorescence (hVEGF_165_). C: Merged figure of A and B illustrating overexpression of both *h*VEGF_165_ and Ang-1 proteins (Scale bar = 50 µm).

Immunofluorescence analyses also showed that both hVEGF_165_ and Ang-1 protein immunofluorescence could be observed in group C. The expressed sites of hVEGF_165_ and Ang-1 protein were located in the cytoplasm rather than in the nucleus (***Fig. 5***).

## DISCUSSION

It is generally recognized that angiogenesis is a complex and multi-gene event. Making therapeutic angiogenesis more closely resemble physiological processes in tissue is extremely important. Thus, multi-angiogenic gene control is technically required to achieve this goal. Physiologically, angiogenesis in tissue relies on two sequential phases including an early phase that strictly depends on the presence of an early angiogenic factor (i.e. VEGF). During the VEGF-dependent period, endothelial cells, mainly originating from the local pre-existing vasculature, become activated to form a set of immature and irregularly shaped vessels surrounded by a thin endothelial layer. This is followed by a late phase where vessel maturation induced by a late angiogenic factor is required to ensure the proper acquisition of functional competence of the newly formed vasculature. During this period, VEGF is not only obsolete but is even detrimental for vessel functionality and pericyte regeneration[16],[17]. Therefore, the approach developed here is an appealing alternative to the simultaneous delivery of VEGF together with other angiogenic factors. Ang-1, one of the late angiogenic factors, has been proven to be capable of promoting angiogenesis and, in synergy with VEGF, maintaining stability and integrity of the mature vasculature by mediating interaction between endothelial cells and their underlying support cells[18]–[21]. Furthermore, Ang-1 is the first angiogenic factor identified to exert a protective effect on the vascular endothelial barrier by blocking the action of permeability-increasing mediators, such as VEGF[8].

Lee *et al*.[22] reported that VEGF and Ang-1 expression was upregulated in human ischemic myocardium. VEGF expression progressively increased over time after the onset of acute ischemia, peaked at 6 weeks and was followed by Ang-1 expression. Therefore, during therapeutic angiogenesis with *VEGF* and *Ang*-1 genes, the accurate and timely control of *Ang*-1 gene expression behind early hypoxia-mediated *VEGF* expression may be a key point for multiple gene-induced angiogenesis in an ischemic heart[23],[24]. So far, an ideal inducible expression control system for *Ang*-1 *in vivo* is the so-called TRE-regulated gene system.

The Tet-On advancedsystem has several advantages over other regulated mammalian gene expression systems: low basal expression, high inducibility, no pleiotropic effects and high absolute expression levels. In our study, we successfully constructed a new Tet-On advanced system for *Ang*-*1* gene inducible expression. We found that, at the cellular level, the Tet-On advanced system effectively activated *Ang*-*1* gene expression with the addition of Dox, a tetracycline (Tc) derivative, to the culture medium and was rapidly turned off upon withdrawal of the antibiotic. Furthermore, Ang-1 expression was tightly regulated dose-dependently in response to varying concentrations of Dox and was independent of the absolute amount of rAAV-TRE-Tight-Ang-1.Dox is a commonly used broad-spectrum antibiotic. In biology, Dox is usually employed in the inducible expression of Tet-on /Tet-Off system or as an inhibitor for suppression of target gene expresssion[25],[26]. Our result suggests that Dox induction was fully necessary for enabling gene control over *Ang*-1 expression. We noted that the background gene expression of *Ang*-1 was extremely low in the absence of Dox due to the combined effect of the modified rtTA and TRE-Tight, while the maximal level of Ang-1 gene expression was comparable with that obtained from strong, constitutive mammalian promoters such as cytomegalovirus (CMV)[27],[28]. This Tet-On advanced system gave us ready access to tightly regulated, high level *Ang*-1 gene expression.

As for rAAV-rtTA-Rs-M2 and rAAV-TRE-Tight-Ang-1, an optimal proportion should meet the need of obtaining the highest level of *Ang*-1 expression with the lowest dose of Dox. We found no significant difference of *Ang*-1 expression in the range of 1:1 to 1:4 (rAAV-rtTA-Rs-M2/rAAV-TRE-Tight-Ang-1). In our recombinant Tet-On advanced system, rtTA-Rs-M2 acted as a control element for Dox. Therefore, under the premise of keeping the highest level of *Ang*-1 expression, our selected ratio of 11:4 guaranteed that the exact amount of rtTA-Rs-M2 was minimized. From the clinical point of view, a low dose of Dox for target gene induction would further decrease possible side effects and improve safety of antibiotic induction.

Concerning the combined efficiency of targeted gene control, *hVEGF*_165_ and *Ang*-1 expression could be induced by altered oxygen and Dox administration, respectively. We noted that one gene control system had no significant interference on the inducible expression of the other target gene, although expression of *hVEGF*_165_ or *Ang*-1 in the co-infected group was lower than that in the single gene infected group.

Based on our results, following early hypoxia-mediated *hVEGF*_165_ expression, it is feasible to realize timely control over *Ang*-1 gene expression with a Tet-On advanced system. Our findings present a strategy of enhanced therapeutic angiogenesis with the sequentially controlled expression of multiple angiogenic genes transferred simultaneously *in vivo*.

Nevertheless, although HRE and the Tet-On advanced systems acted as effective double gene switches *in vitro*, our new system needs to be validated in animal models in which actual ischemic myocardial pathologies can be reproduced. Additionally, the combined efficiency of VEGF and Ang-1-driven therapeutic angiogenesis should be further evaluated.

In conclusion, HRE and the Tet-On advanced systems have been verified to be new promising gene switches for *VEGF* and *Ang*-1-inducible expression control *in vitro*. Thus, this innovative double gene control system leads to a broader prospect for effective and safe angiogenic therapy in ischemic heart disease *in vivo*.
